# Heat Shock Proteins 60 and 70 Specific Proinflammatory and Cytotoxic Response of CD4^+^CD28^null^ Cells in Chronic Kidney Disease

**DOI:** 10.1155/2013/384807

**Published:** 2013-11-21

**Authors:** Ashok K. Yadav, Vinod Kumar, Vivekanand Jha

**Affiliations:** ^1^Department of Nephrology, Postgraduate Institute of Medical Education and Research, Sector 12, Chandigarh 160 012, India; ^2^George Institute of Global Health, Splendor Forum, Jasola, New Delhi 110 025, India

## Abstract

*Background*. CD4^+^CD28^null^ T cells are expanded in peripheral blood of patients with chronic kidney disease and associated with subclinical atherosclerosis. However, triggers for the oligoclonal expansion and activation of these cells are not clear. *Methods*. We investigated twenty-five stage V-IV chronic kidney disease (CKD) patients and eight healthy subjects (HC). Peripheral mononuclear cells were isolated and incubated with heat shock protein- (HSP) 60 and 70. CD4^+^CD28^null^ and CD4^+^CD28^+^ cells were sorted by flowcytometry and antigen specific response was assessed by the mRNA and protein expression of interferon (IFN)-*γ*, perforin, and granzyme B using qRT-PCR and Elispot. *Results*. The basal mRNA expression of IFN-*γ*, perforin, and granzyme B in CD4^+^CD28^null^ cells was higher in subjects with CKD compared to that in HC (*P* < 0.0001). Subjects with CKD also showed expression of IFN-*γ*, perforin, and granzyme B in the CD4^+^CD28^+^ subset, but this was much weaker than that seen in the CD4^+^CD28^null^ population (*P* < 0.0001). We did not note the expression of these molecules at mRNA or protein level in either subset of CD4 cells in HC. After incubation with HSP60 and HSP70, CD4^+^CD28^null^ cells showed increased expression at mRNA (*P* < 0.001) and protein level (*P* < 0.001). CD4^+^CD28^+^ cells also showed a weak increase in expression. No antigen-specific response was noted in HC. *Conclusion*. These data show that CD4^+^CD28^null^ cells in subjects with CKD react with HSP60 and HSP70 by upregulating the expression of IFN-*γ*, perforin and granzyme B. Increased circulating level of HSP60 and HSP70 might play a role in initiation and/or progression of atherosclerosis in CKD subjects through perturbation of CD4^+^CD28^null^ cells.

## 1. Introduction

Patients with chronic kidney disease (CKD) exhibit an expansion in the circulating CD4^+^CD28^null^ cell population [1, 2]. In contrast to the classical CD4^+^ T helper cells, cells lacking CD28 molecule are cytotoxic and proinflammatory [3–5]. The ability of CD4^+^CD28^null^ cells to interact with the endothelial cells through fractalkine-CX3CR interaction and the demonstration of these cells in atherosclerosis plaque, in combination with their ability to produce high level of IFN-*γ* and cytotoxic molecules leading to plaque destabilization, suggests their role in atherosclerotic disease [6]. The exact mechanism of their activation and expansion, however, is unclear. We have shown that this population is expanded in CKD even before development of clinically overt atherosclerotic coronary artery disease (CAD), and the degree of expansion correlates with common carotid artery intima media thickness, a surrogate marker of atherosclerosis [2, 6]. 

In the early phases of atherogenesis, T-cell recruitment is mediated by nonspecific inflammatory signals and is antigen-independent. Later, the expansion of the CD4^+^CD28^null^ T subset becomes oligoclonal, suggesting repetitive antigenic stimulation by a limited number of antigens [7–9]. Results obtained from animal models that allow detailed characterization of T-cells isolated from atherosclerotic lesions in different stages of disease support this hypothesis [10].

Betjes et al. recently showed that latency for *cytomegalovirus *(CMV) is an independent risk factor for atherosclerosis in patients with ESRD [11]. In addition, analysis of the T-cell subsets indicated that numbers of CMV-specific T-cells increased as the stage of renal failure progressed [12]. CD4^+^ T lymphocytes also recognize proteins derived from other microorganisms such as *Chlamydia pneumoniae*, *Herpes simplex*, *Helicobacterpylori*, and CMV [13–16].

In contrast to the findings of Betjes et al., the association between CMV positivity and CD4^+^CD28^null^ cell population was not confirmed in other studies [5, 17, 18], raising the possibility that other antigenic stimuli might contribute to the expansion of these cells. Candidates include endogenous antigens that lead to antigen-driven T-cell proliferation upon chronic exposure, explaining the finding of oligoclonal expansion of antigen specific CD4^+^CD28^null^ T-cells.

Endogenous antigens like oxidized low-density lypoproteins (oxLDL) and heat shock proteins (HSP) have been implicated in atherogenesis [14, 19, 20]. These antigens are expressed in atherosclerotic plaques and their levels correlate with the severity of atherosclerosis. T-cells specific for oxidized oxLDL and HSPs have been isolated from atherosclerotic plaques [14, 20, 21]. About 50% of the CD4^+^CD28^null^, T-cell clones derived from patients with acute coronary syndrome recognize HSP60, and when stimulated with autologous HSP60, CD4^+^CD28^null^ T-cell clones produce IFN-*γ* and perforin [22]. Similarly, HSP70 has been detected in both fibrotic and necrotic atherosclerotic plaques [23]. Chan et al. demonstrated a positive correlation between anti-HSP70 antibodies and vascular diseases indicating that HSP70 and anti-HSP 70 are involved in the pathogenesis and propagation of atherosclerosis [24]. A recent study shows that HSP70 can induce cytolytic activity of T-helper cells and that the release of granzyme B is target-independent [25].

In the present study we investigated the antigen specific effect of HSP60 and HSP70 on CD4^+^CD28^null^ cells in patients with CKD, using perforin, granzyme, and IFN-*γ* as markers of activation. Antigen-specific response of these cells may provide insight into a possible contributory pathway of atherosclerosis development and/or progression in CKD.

## 2. Materials and Methods

Twenty-five nondialysis stage 4-5 CKD subjects without CAD, who exhibited significant increase (>15%) in the CD4^+^CD28^null^ cell population were selected [2].  Table 1 shows the demographic and clinical profile of these subjects. We also investigated 8 healthy individuals with normal renal function and no history of coronary artery disease, hypertension, or diabetes. 

### 2.1. Peripheral Mononuclear Cell Separation and Antigen Stimulation

Peripheral blood mononuclear cells (PBMCs) were separated by density gradient centrifugation using ficolhistopaque (Sigma-Aldrich). PBMCs (4 × 10^6^ cells/mL) were incubated with recombinant human HSP60 (5 ng/mL) and HSP70 (5 ng/mL) expressed in *Escherichia coli* (>95% purity on SDS-PAGE) to assess the antigen specific response in RPMI 1640 medium supplemented with 10% fetal bovine serum, 2 mM L-glutamine, 50 units/mL penicillin, and 50 units/mL streptomycin (all from Sigma-Aldrich) for 48 hours in CO_2_ incubator at 37°C. Phytohemagglutinin (PHA) served as the positive control (10 *μ*g/mL). After 48 hours, cells were washed and CD4^+^CD28^null^ and CD4^+^CD28^+^ cells were sorted by FACS.

### 2.2. Cell Sorting

CD4^+^CD28^null^ and CD4^+^CD28^+^ cells were separated by FACS Aria II sorter. PBMCs (4 × 10^6^ cells) were stained with anti-CD4 and anti-CD28 antibodies and incubated for 30 min at 4°C in dark. Stained cells were washed twice with phosphate buffered saline (PBS) containing 1% fetal bovin serum (FBS) and resuspended in 1x PBS + 1% FBS. Sorting was done using double discriminating gating. The efficiency of the sorting was 90–95%. Postsort analysis showed the purity of the sorted cell to be 96–99%. The postsort analysis also confirmed the viability of sorted cell >92%.

### 2.3. mRNA Analysis

Total RNA from sorted cells was isolated using Ambion Micro kit and cDNA was synthesized using the RevertAid First strand cDNA Synthesis kit (Fermentas Life Sciences, USA) according to manufacturer's instructions. mRNA analysis of IFN-*γ*, perporin and granzyme B was done with Taqman gene expression assay (Life Technologies) by relative quantification method on real-time PCR machine (ABI Biosystem Prism 7500). *β*-actin was used as internal control. Relative expression was compared between CD4^+^CD28^null^ and CD4^+^CD28^+^ cells in CKD as well as healthy subjects in both unstimulated and stimulated states.

### 2.4. ELISPOT Assay

**Figure 1:**
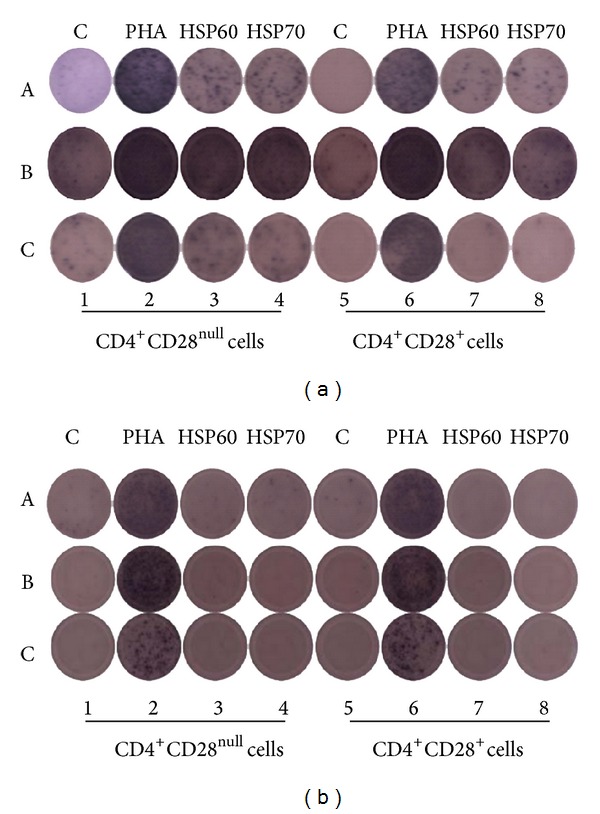
Representative image of ELISPOT analysis in patients with CKD (a) and healthy subjects (b): [1–4] CD4^+^CD28^null^ cells and [5–8] CD4^+^CD28^+^ cells stimulated with and without PHA (10 *μ*g/mL), HSP60 (5 ng/mL), and HSP70 (5 ng/mL), respectively. (A) Interferon-*γ* spots, (B) perforin spots, and (C) granzyme B spots. CD28^null^ cells responded strongly to HSP60 and HSP70 as compared to CD28^+^ cells as shown by the spot development for IFN-*γ*, perforin, and granzyme B in CKD subjects whereas there was no response as shown by lack of spot development for IFN-*γ*, perforin, and granzyme B in healthy controls. However, the cells responded robustly to PHA, a positive control as shown by the interferon-*γ*, perforin, and granzyme B spots.

Elispot assay was done to analyze the antigen-specific expression of IFN-*γ*, perforin, and granzyme B. Elispot kit from Millipore was used for this assay as per their protocol. Briefly, ninety-six-well Elispot plates were used for each molecule. Wells were prewetted with 70% ethanol for 2 min and washed 5 times with 1x PBS. Capture antibody in the concentration of 15 *μ*g/mL for IFN-*γ* and granzyme B and 30 *μ*L/mL for perforin were used for coating the wells overnight at 4°C. After incubation, plates were washed 5 times with 1x PBS; 200 *μ*L RPMI1640 medium was added to each well and incubated for two hours at 37°C in CO_2_ incubator. Medium was discarded and 2 × 10^4^/well sorted CD4^+^CD28^null^ and CD4^+^CD28^+^ were stimulated with and without HSP60, HSP70 (5 ng/mL each), and PHA (10 *μ*g/mL) for 48 hours at 37°C in CO_2_ incubator. After 48 hours, medium was discarded and plates were washed 5 times with 1x PBS. Biotinylated detection antibodies (1 *μ*g/mL) were added to the wells and incubated for two hours. Plates were washed 5 times with 1x PBS and streptavidin-alkaline phosphatase conjugate (1 *μ*g/mL) was added and incubated for 1 hour. After washes, 100 *μ*L nitro-blue tetrazolium and 5-bromo-4-chloro-3′-indolyphosphate (BCIP-NBT) substrate were added for the spot development. Development was stopped by washing with tap water. Plates were dried and the spots were counted on CTL Elispot counter (Cellular Technology Ltd., Cleveland, Ohio, USA). Representative Elispots are shown in Figure 1.

#### 2.4.1. Statistics

Results were presented as mean ± SEM for continuous variables and as percentages for categorical data. Significant differences between groups were analyzed using Student's *t*-test. Pair wise *t*-test was used within groups. Differences were considered to be significant when two tailed *P* was less than 0.05.

## 3. Results

### 3.1. Transcript Level Analysis

**Figure 2:**
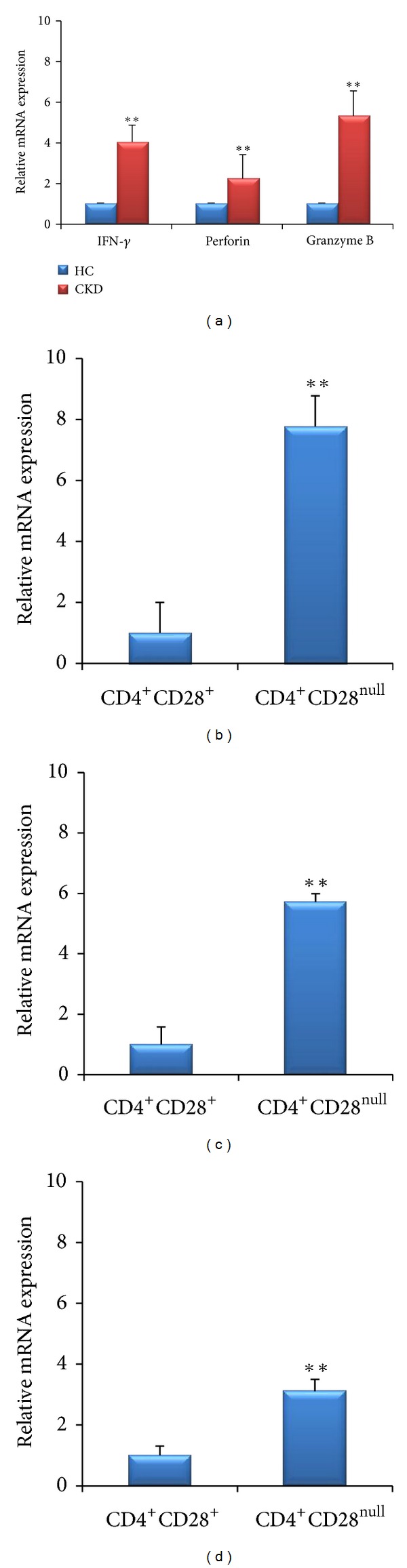
mRNA expression of (a) IFN-*γ*, perforin, and granzyme B in CD4^+^CD28^null^ cells in CKD and HC; ((b)–(d)) IFN-*γ*, perforin, and granzyme B in CD4^+^CD28^+^ and CD4^+^CD28^null^ T-cells, respectively, in CKD subjects. **P < 0.0001.

Basal expression of IFN-*γ*, perforin, and granzyme B in CD4^+^CD28^null^ cells was significantly more in CKD as compared to HC (*P* < 0.0001, 0.008, and *P* < 0.0001, resp.) (Figure 2(a)).Compared to CD4^+^CD28^+^ T-cells, the expression of IFN-*γ*, perforin, and granzyme B was higher in CD4^+^CD28^null^ T-cells in CKD subjects (*P* < 0.0001, <0.0001, and <0.0001, resp.) (Figures 2(b)–2(d)). Very weak expression of IFN-*γ* and no detectable expression of perforin and granzyme B were noted in CD4^+^CD28^+^ cells in HC.

**Figure 3:**
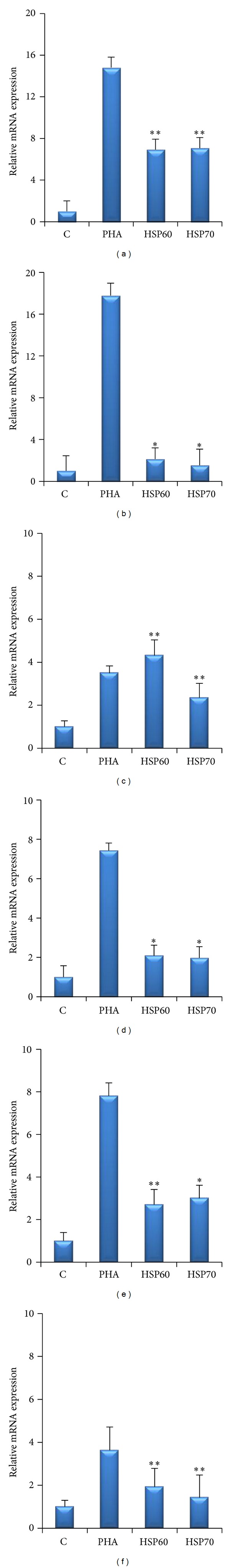
Relative mRNA expression of IFN-*γ*, perforin, and granzyme B in CD4^+^CD28^null^ cells ((a), (c), (e)) as well as in CD4^+^CD28^+^ cells ((b), (d), (f)) in CKD subjects. 48 hr of stimulation of HSP60 (5 ng/mL) and HSP70 (5 ng/mL) increased the expression of IFN-*γ*, perforin, and granzyme B in both T-cell subsets compared to unstimulated controls. PHA (10 *μ*g/mL) served as a positive control for both T-cell subsets. **P < 0.0001, *P < 0.001.

**Figure 4:**
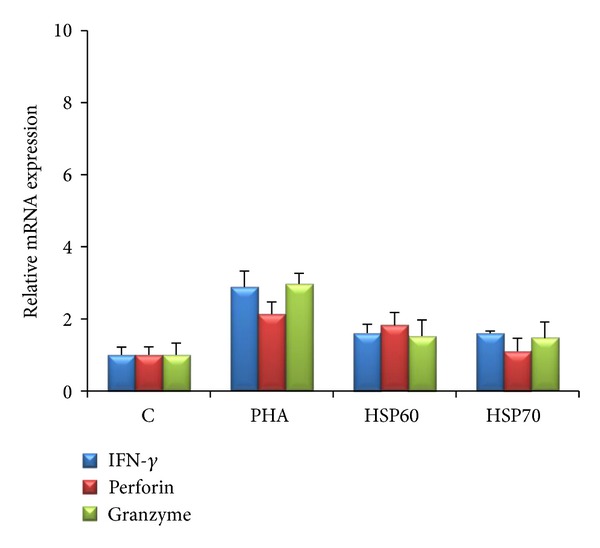
Relative mRNA expression of INF-*γ*, perforin, and granzyme B in CD4^+^CD28^null^ cells in healthy subjects. No antigenic response was observed in cells stimulated with HSP60 (5 ng/mL) and HSP70 (5 ng/mL), whereas PHA (10 *μ*g/mL) (positive control) showed increased expression of mRNA.

Further, incubation with HSP60 and HSP70 increased the expression of IFN-*γ* (*P* = 0.004 and *P* = 0.002), perforin (*P* = 0.0006 and *P* = 0.002), and granzyme B (*P* = 0.02 and *P* = 0.009) in CD4^+^CD28^null^ T-cells in CKD subjects (Figure 3). An increase in expression of IFN-*γ* (*P* = 0.02 and *P* = 0.01), perforin (*P* = 0.03 and *P* = 0.01), and granzyme B (*P* = 0.0004 and *P* < 0.0001) was also noted in CD4^+^CD28^+^ T-cell subset in CKD subjects. In contrast, cells isolated from HC did not show any increase in expression over the basal values (Figure 4). PHA caused an increase in the transcript level of responder molecules both in CD4^+^CD28^null^ and CD4^+^CD28^+^ T-cells in CKD as well as in HC.

### 3.2. ELISPOT Analysis

**Figure 5:**
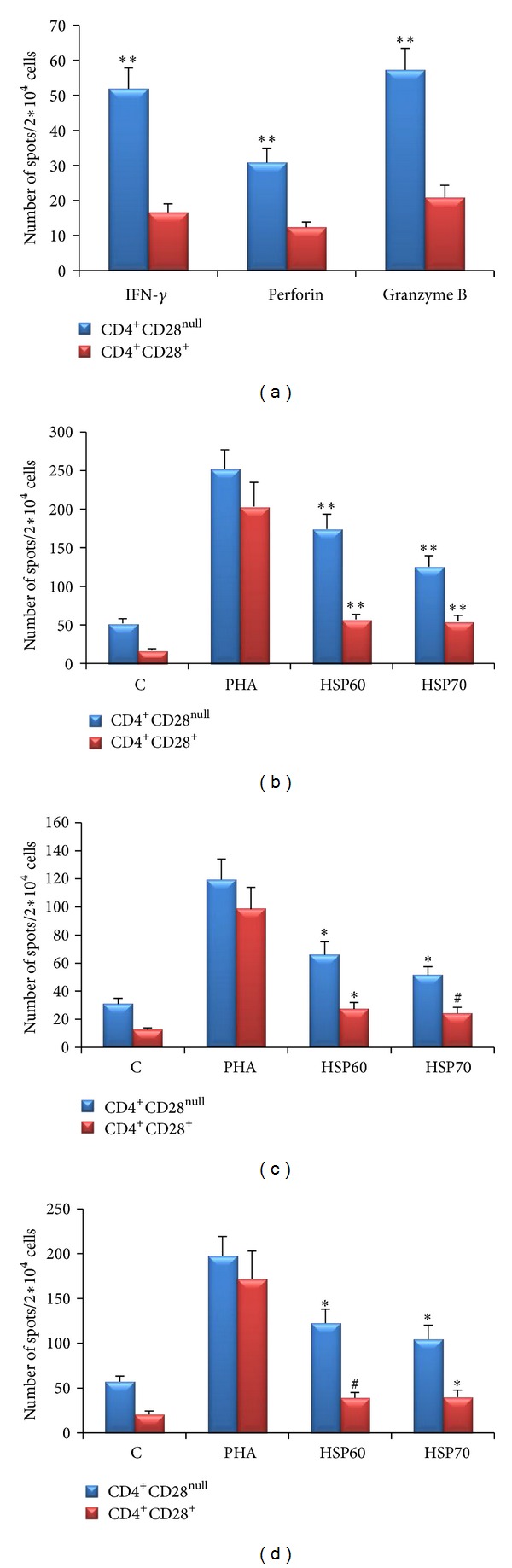
Elispot counts of IFN-*γ*, perforin, and granzyme B in CD4^+^CD28^null^ cells and CD4^+^CD28^+^ cells in CKD subjects. (a) Unstimulated cells: expression of IFN-*γ*, perforin, and granzyme B was significantly higher in CD4^+^CD28^null^ T-cells. Elispot counts of (b) IFN-*γ*, (c) perforin and (d) granzyme B were significantly increased after 48 hr stimulation with HSP60 (5 ng/mL) and HSP70 (5 ng/mL) as compared to unstimulated control in CD4^+^CD28^null^ cells as well as CD4^+^CD28^+^ cells. PHA (10 *μ*g/mL) served as positive control. **P < 0.0001, *P < 0.001, ^#^ *P* < 0.01.

The basal levels of IFN-*γ*, perforin and granzyme B were higher in CD4^+^CD28^null^ T-cells as compared to CD4^+^CD28^+^ cells (Figure 5(a)) in patients with CKD (*P* < 0.0001, *P* = 0.0002, and *P* < 0.0001, resp.). The trend of antigen specific response was similar to that seen on transcript analysis. Incubation with HSP60 and HSP70 was followed by increase in the expression of IFN-*γ* (*P* < 0.0001 and *P* < 0.0001), perforin (*P* = 0.002 and *P* = 0.009) and granzyme B (*P* < 0.0001 and *P* = 0.005) in CD4^+^CD28^null^ cells. Similar trend was noted in CD4^+^CD28^+^ T-cells after stimulation with HSP60 and HSP70 (IFN-*γ*; *P* < 0.0001 and *P* < 0.0001, perforin; *P* = 0.008 and *P* = 0.02, and granzyme B; *P* = 0.01 and *P* = 0.002) (Figures 5(b)–5(d)). As noted in transcript analysis, cells from healthy controls did not exhibit significant secretion of either of these molecules (spot count < 10) in either T-cell subset in either basal state or after stimulation with HSP60 and HSP70. The response to PHA confirms the accuracy of the experiments (Figure 4). The number of spots counted in CD4^+^CD28^null^ and CD4^+^CD28^+^ T-cells in stimulated and unstimulated cells are shown in Table 2.

## 4. Discussion

CKD is associated with abnormalities in various biological pathways that culminate in complications related to almost all organ systems. Most complications are multifactorial in origin, with potential culprits being uremic toxins, inflammatory mediators, reactive oxygen species, apoptosis, and infections. Initiation of dialysis imposes further stress on already dysregulated pathways. 

Cardiovascular abnormalities have emerged as the major threat to CKD patients, and the role of CD4^+^CD28^null^ T-cells has come under scrutiny. However, the reasons behind expansion of CD4^+^CD28^null^ T-cells remain unclear. The most widely accepted hypothesis is that repeated antigenic stimulation leads to oligoclonal expansion and inflammatory activation of these cells. 

The mechanism of emergence and growth of these cells is unknown. Chronic infections and markers of inflammation have been shown to be associated with atherosclerosis [26]. Studies in rheumatoid vasculitis have shown a decline in the two nuclear protein binding motifs *α* and *β* within the CD28 minimal promoter, suggesting that these cells emerge from chronic persistent stimulation of CD4^+^CD28^+^ progenitors, as opposed to CD28 loss by replicative senescence, in which only *β*-binding promoter motif is downregulated [27]. Evidence to support the role of antigenic stimulation in exacerbated loss of the CD28 expression has also been presented in infections with the human immunodeficiency virus as well as *Trypanosoma cruzi* in Chagas's disease [28–30].

Whether these antigens are exogenous, such as of microbial origin [13–16], or endogenous, like oxLDL and HSPs, remains a matter of conjecture [19–21]. It is possible that all these stimulants work in different situations. Betjes et al. have shown the relationship between CMV serostatus and number as well as activation of these cells. Seropositive ESRD patients over the age of 50 years showed a 50-fold increase in CD4^+^CD28^null^ cells as compared to CMV seronegative ESRD and 5-fold as compared to CMV seropositive HC. Further, upon CMV stimulation these cells showed increased degranulation and IFN-*γ* secretion indicating their cytotoxic and proinflammatory character [1]. Other studies, however, failed to find increased reactivity of CD4^+^CD28^null^ cells with CMV and other antigens such as *Chlamydia pneumoniae* and ox-LDL [17, 18, 22]. Therefore, alternative explanations are likely, especially in geographic areas with different rates CMV infection. CMV seropositivity is universal in India. 

HSPs, primarily regarded solely as intracellular chaperones, have been shown to have immunomodulatory activity, especially when present outside the cells, and have been implicated in atherogenesis [31]. HSP60 and HSP70 are considered to be stress-induced isoforms which leak outside the cells either by active transport or after cell death and stimulate cytokine production and adhesion molecule expression on endothelial cells [32]. Since CKD is a proinflammatory condition, characterized by release of a number of stress molecules, it is conceivable that HSPs are involved in the pathogenesis of complications of CKD [33]. 

Demonstration of increased expression of IFN-*γ*, perforin and granzyme B in CD4^+^CD28^null^ cells at both mRNA level as well as protein level in CKD subjects, while cells from healthy subjects showed no such response, suggests that an autoimmune T-cell-mediated response may hold the balance. Although previous studies have reported the presence of HSP-reactive T-cells within atheromatous lesions [15] and in CAD [18], this is the first investigation into antigenic specificity of the CD4^+^CD28^null^ cells in CKD subjects. 

Release of HSP60 in the absence of cell necrosis triggers an immune response. Human HSP60 has been shown to activate a proinflammatory reaction in innate immune cells. Incubation of dendritic cells with HSP60 triggered the release of TNF-*α*, IL-6, and other chemokines [34–36]. In our previous study [2] we showed that there was a positive association between CD4^+^CD28^null^ cells and serum TNF-*α* level and also TNF-*α* downregulated CD28 expression. This explains to some extent that HSP triggered TNF-*α* release plays a role in generation of CD4^+^CD28^null^ cells. 

Apart from HSP60 and HSP70, HSP90 also participates in the immune response [37]. *In vitro*, HSP90 activated Th1 and Th2 lymphocytes and stimulated IFN-*γ* and IL-4 production [38]. In a recent study, HSP90 expression was associated with features of plaque instability in advanced human lesions of atherosclerosis [39]. 

In our study, CD4^+^CD28^+^ also showed reactivity to both HSP60 and HSP70, but to a lesser extent than CD4^+^CD28^null^ cells. Responsiveness of the CD4^+^CD28^+^ population in CKD subjects confirms previous exposure of these patients to the antigens used. It is possible that *in vivo* repeated HSP stimulation of the CD4^+^CD28^+^ cells in CKD patients might account for the loss of the CD28 marker and emergence of the CD4^+^CD28^null^ cells. Worth noting is the absence of any response in either subset of CD4^+^ T-cells in healthy subjects. One of the reasons may be the low level of circulating HSPs in healthy subjects, and *in vitro *stimulation did not affect as the cells had not encountered these proteins previously.

In conclusion, our findings point towards a potential role of endogenous HSP60 and HSP70s in the downregulation of CD28 molecule from the classical CD4 cells which makes them exhibit an effector phenotype in CKD.

## Figures and Tables

**Table 1:** Demographic and biochemical characteristics of the subjects.

	CKD patients	Healthy Controls
Number of cases	25	8
Age (years)	53.1 ± 2.4	48.3 ± 7.3
Gender M/F	16/7	5/3
Body mass index (kg/m^2^)	22.8 ± 0.5	21.9 ± 2.0
Systolic blood pressure (mm Hg)	136.7 ± 2.8	116.1 ± 2.3
Diastolic blood pressure (mm Hg)	84.3 ± 1.3	78.4 ± 1.9
Hemoglobin (mg/dL)	10.75 ± 0.7	13.5 ± 2.1
Serum urea (mg/dL)	115.8 ± 8.3	
Serum creatinine (mg/dL)	6.4 ± 0.7	0.9 ± 0.02
Serum albumin (g/dL)	4.02 ± 0.20	
Total cholesterol (mg/dL)	162.6 ± 8.7	134.1 ± 7.5
Triglycerides (mg/dL)	150.8 ± 23.0	
Calcium (mg/dL)	8.1 ± 0.3	
Inorganic phosphate (mg/dL)	6.5 ± 0.5	
CD4^+^CD28^null^ cells (%)	20.4 ± 1.6	5.6 ± 2.1

**Table 2:** IFN-*γ*, perforin and granzyme B secreted (numbers of spots) from CD4^+^CD28^null^ cell and CD4^+^CD28^+^ T cells during ELISPOT assay with and without stimulation with PHA (10 *µ*g/mL), HSP60 (5 ng/mL) and HSP70 (5 ng/mL) in CKD subjects.

	CD4^+^ Cells	Unstimulated Control	PHA	HSP60	HSP70
IFN-*γ*	CD28^null ^	51.92 ± 5.9	252.56 ± 24.5	174.32 ± 19.3	125.48 ± 14.3
	CD28^+^	16.64 ± 2.4	203.44 ± 31.3	57.12 ± 6.4	54.64 ± 7.7
Perforin	CD28^null^	30.84 ± 4.1	119.44 ± 14.7	65.92 ± 9.3	51.4 ± 6.1
	CD28^+^	12.36 ± 1.5	98.44 ± 15.5	27.24 ± 4.7	24.08 ± 4.4
Granzyme B	CD28^null^	57.28 ± 6.2	197.52 ± 21.8	122.52 ± 15.6	104.56 ± 15.8
	CD28^+^	20.8 ± 3.5	171.6 ± 31.3	39.2 ± 5.9	40 ± 7.6
